# Clinical outcomes of newly diagnosed PCNSL treated with rituximab-methotrexate-cytarabine with or without ibrutinib: a retrospective study

**DOI:** 10.3389/fimmu.2025.1579483

**Published:** 2025-05-22

**Authors:** Wenhua Wang, Bingyi Wang, Yifei Sun, Lihua Qiu, Zhengzi Qian, Shiyong Zhou, Zheng Song, Wei Li, Xudong Zhang, Lanfang Li, Xianhuo Wang, Huilai Zhang

**Affiliations:** ^1^ State Key Laboratory of Druggability Evaluation and Systematic Translational Medicine, Department of Lymphoma, Tianjin Medical University Cancer Institute and Hospital, National Clinical Research Center for Cancer, Tianjin’s Clinical Research Center for Cancer, Tianjin, China; ^2^ Key Laboratory of Cancer Prevention and Therapy, The Sino-United States Center for Lymphoma and Leukemia Research, Tianjin, China; ^3^ Department of Oncology, The First Affiliated Hospital of Zhengzhou University, Zhengzhou, Henan, China

**Keywords:** central nervous system, lymphoma, ibrutinib, retrospective, methotrexate

## Abstract

**Objective:**

This study aimed to evaluate the efficacy and safety of rituximab, methotrexate, cytarabine with or without ibrutinib in newly diagnosed primary central nervous system lymphoma (PCNSL) and explore the correlation between efficacy and genomic alterations.

**Methods:**

From March 2013 to October 2022, data from 88 patients with newly diagnosed PCNSL were retrospectively collected and analyzed. Fifty-nine patients received rituximab, methotrexate and cytarabine (RMA, group A), and twenty-nine patients received the same RMA combined with ibrutinib (RMA + Ibrutinib, group B).

**Results:**

At a median follow-up of 27.7 months, the complete response rate (CRR), overall response rate (ORR) and overall survival (OS) in group B superior to group A (41.4% versus 16.9% for CRR, P=0.013; 86.2% versus 59.3% for ORR, *P*=0.011; P=0.036 for OS). The ORR, progression-free survival (PFS) and OS of RMA + ibrutinib +deep lesions (group C) were better than those of RMA + deep lesions (group D) (*P*=0.027 for ORR, *P=*0.046 for PFS, *P*=0.004 for OS). Patients in group B had no more toxicities than those in group A and the most common adverse events in the two groups were primarily grade 1-2. Sequencing of tumor tissues from 22 patients showed that *MYD88* mutations were the most frequent genetic alterations, two patients with *CARD11* mutation did not respond to treatment and three patients without an *MYD88* or *CD79B* had response after treatment.

**Conclusions:**

RMA in combination with ibrutinib regimen improved response rates and survival in newly diagnosed PCNSL with no serious adverse effects. Mutations in *CARD11* gene may provide directions for patients to select targeted drugs.

## Introduction

Primary central nervous system lymphoma (PCNSL) is an extranodal non-Hodgkin lymphoma that occurs in the brain, leptomeninges, spinal cord, central nerves and eyes (1). Most PCNSL tumors are nongerminal center B-cell-like (non-GCB) subtypes of diffuse large B-cell lymphoma (DLBCL) (2, 3). PCNSL accounts for less than 1% of all lymphomas, 3% of all central nervous system (CNS) tumors and 4%-6% of all extranodal lymphomas. Immunodeficiency is the primary risk factor for the occurrence of PCNSL (4). In recent years, the incidence of this disease has increased, especially in elderly individuals (5, 6). The treatment of PCNSL includes induction and consolidation therapy, and there are no standard regimens. Multidrug chemotherapy based on high-dose methotrexate (HD-MTX) is usually deemed the standard induction method. Consolidation regimens include autologous stem cell transplantation (ASCT) and whole-brain radiotherapy (WBRT) (7–10). Although therapeutic progress for PCNSL has been achieved, 15%-25% of patients have refractory disease, and 25%-50% of patients relapse after initially having a response (11–13). Notably, elderly individuals are more likely to relapse than others, although age is not a factor in a poor prognosis (14).

Bruton’s tyrosine kinase (BTK) is the crucial component linked with the B-cell antigen receptor (BCR), Toll-like receptor (TLR) and nuclear factor kappa B (NF-κB) signaling pathways. Mutations in myeloid differentiation primary response 88 (*MYD88*) and *CD79B* activate the BCR, TLR and NF-κB signaling pathways, disturb the cell cycle, facilitate immune escape, and inhibit B-cell apoptosis (15–17). Compared with systemic DLBCL, alterations in BCR signaling pathways occur more frequently in PCNSL (18). Therefore, BTK is an attractive treatment target for PCNSL. Ibrutinib, a first-in-class BTK inhibitor, has activity in refractory/relapsed (R/R) PCNSL through reducing NF-κB pathway activity (18). Studies have suggested that Ibrutinib has potential efficacy in R/R PCNSL patients (18–20). In addition, ibrutinib has been included in the National Comprehensive Cancer Network (NCCN) guidelines for R/R PCNSL treatment. However, studies on ibrutinib in patients with newly diagnosed PCNSL are rare. In this study, we retrospectively compared and analyzed the efficacy and safety of rituximab-methotrexate-cytarabine with or without ibrutinib in newly diagnosed PCNSL patients to explore whether ibrutinib is beneficial for the first-line treatment of PCNSL and to explore the correlation between efficacy and genomic alterations.

## Materials and methods

### Patients and treatment

**Figure 1:**
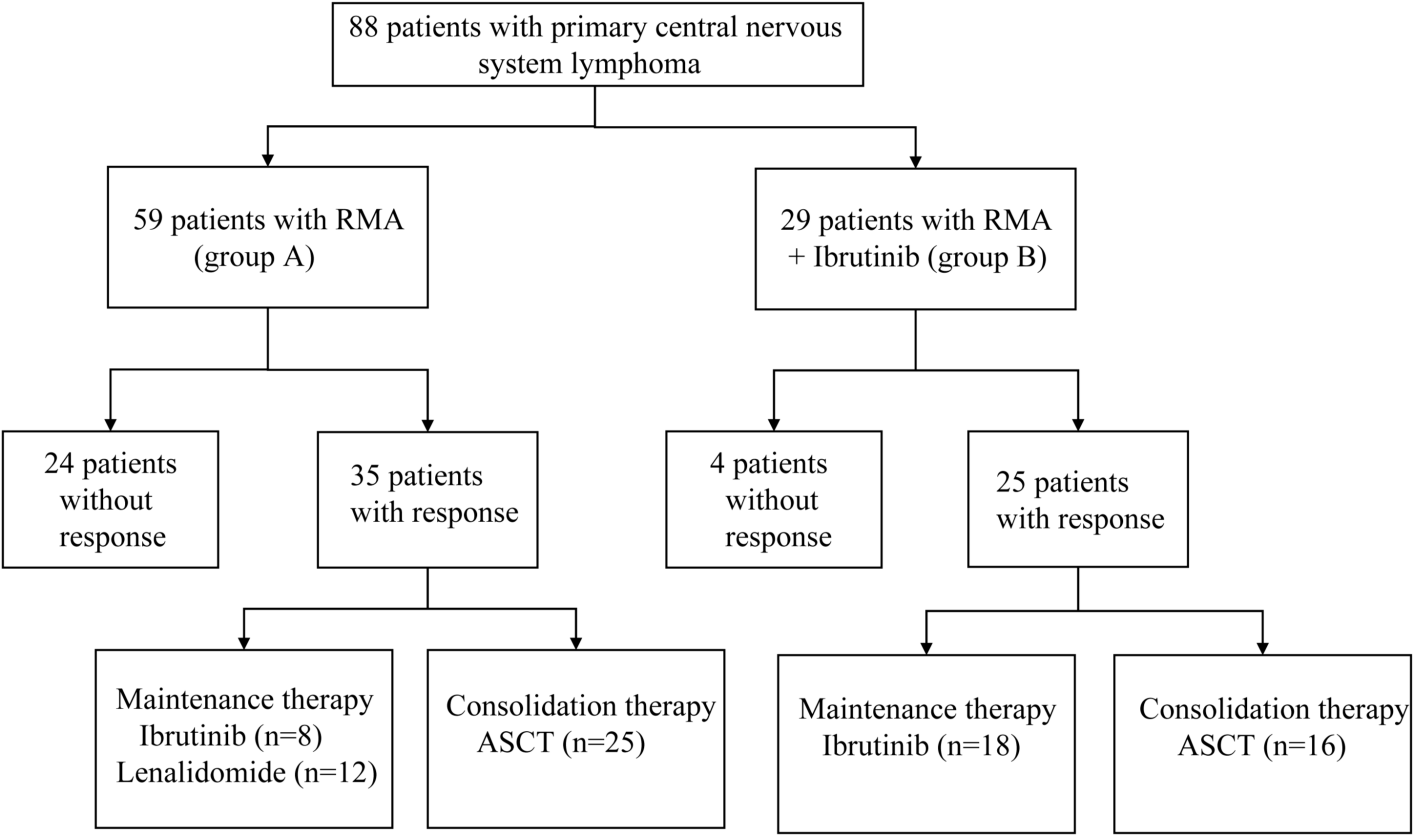
This study profile.

From March 2013 to October 2022, 88 newly diagnosed PCNSL patients from Tianjin Medical University Cancer Institute & Hospital (TMUCIH) and the First Affiliated Hospital of Zhengzhou University were retrospectively enrolled and analyzed. Eighty-eight patients received the two study regimens: rituximab 375 mg/m² (intravenous infusion) on day 0, methotrexate 3.5 g/m² (0.5 g/m² in 15 min, followed by 3 g/m² in a six h infusion) on day 1 and cytarabine 1.0 g/m^2^ (1 h infusion, every 12 h) on days 2–3 every 28 days, for four cycles in total (RMA; group A), or the same rituximab-methotrexate-cytarabine combined with ibrutinib (560 mg/d) (RMA + Ibrutinib; group B). Ibrutinib was suspended on HD-MTX infusion days and restarted after HD-MTX clearance. Ibrutinib was administered each day uninterruptedly after induction therapy until intolerable toxicity, disease progression or death occurred. After induction therapy, patients received consolidation therapy including thiotepa-containing conditioning regimen and ASCT, followed by maintenance therapy with ibrutinib (560mg/d) or lenalidomide (10mg/d). We defined groups C, D, E and F: group C = deep lesions in R-MA; group D = deep lesions in R-MA + ibrutinib; group E = multiple lesions in R-MA; group F = multiple lesions in R-MA+ ibrutinib. **Figure 1** shows the study flowchart. The study was approved by the institutional review board of the Tianjin Medical University Cancer Institute & Hospital and the First Affiliated Hospital of Zhengzhou University.

### Assessment of efficacy and adverse events

The efficacy of treatment was evaluated according to the International PCNSL Collaborative Group (IPCG) Response Criteria (21). The response was identified by changes in tumor volume on MRI every two cycles and cerebrospinal fluid (CSF) cytology. The best response after treatment was assessed, and the overall response rate (ORR) was calculated, in which the ORR was defined as the sum of patients with a complete response (CR; the disappearance of all lymphoma diseases) and patients with a partial response (PR; 50% or more significant reduction in tumor volume). The total tumor volume was the sum of the disease volume (6 or fewer CNS compartments) calculated by its maximum longitudinal diameter multiplied by its vertical diameter on the same MRI scan. Adverse events (AEs) were recorded via physical examination; laboratory tests such as a hematological panel, plasma biochemical panel and electrocardiograms; and classification of AEs was made following the National Cancer Institute Common Terminology Criteria for Adverse Events (CTCAE, version 5.0) (22).

### Sample collection

Baseline tumor samples from 22 patients were analyzed by targeted sequencing of a 307 lymphoma-associated panel. The non-GCB and germinal center B-cell-like (GCB) *Hans’s* classification determined subtype.

### Statistical analysis

SPSS25 (IBM, Chicago, IL) and R statistical programming environment (v4.0; The R Project for Statistical Computing, Vienna, Austria) software were used for statistical analyses. The χ² test or Fisher’s exact test was used to compare characteristics and response rates between therapeutic groups. Mann–Whitney tests were used to compare quantitative and ordinal variables. Progression-free survival (PFS) was calculated as the time from diagnosis to disease progression, death or last follow-up. Overall survival (OS) was calculated as the time from the diagnosis to death or the last follow-up. Survival curves were generated via the Kaplan–Meier method, and comparisons were performed via the log-rank test. All *P* values were two-sided, and *P*<0.05 was considered a significant difference.

## Results

### Patient population

The clinical and pathological characteristics of the RMA (group A) and RMA + Ibrutinib (group B) groups are shown in **Table 1**. The median age of the 88 patients was 59 (19-82) years, and 40 (45.5%) patients were male. Forty-two (47.7%) patients had an Eastern Cooperative Oncology Group (ECOG) performance status greater than or equal to 2. Twenty (22.7%) and 12 (13.6%) patients had elevated lactate dehydrogenase (LDH) and β2-microglobulin (β2-MG) levels, respectively. Thirty-four (38.6%) patients had multiple lesions at the first registration, and 47 (53.4%) patients had lesions in deep intracranial areas. The Memorial Sloan−Kettering Cancer Center (MSKCC) risk score was low for 20 (22.7%) patients and high for 14 (15.9%). Sixty-three (71.6%) patients were diagnosed with the non-GCB subtype. Group B had more patients with multiple lesions at the first registration than in group A (*P*=0.007). The groups were well-balanced in terms of patient characteristics, such as age, sex, and ECOG PS, none of which differed significantly between group A and group B.

**Table 1:** Baseline characteristics of patients in the group A and group B.

Characteristics	RMA (group A, n=59)	RMA+ Ibrutinib (group B, n=29)	*p* Value
Age (years)			0.113
≤60	35 (59.3)	12 (41.4)	
>60	24 (40.7)	17 (58.6)	
Sex			0.590
Male	28 (47.5)	12 (41.4)	
Female	31 (52.5)	17 (58.6)	
ECOG PS			0.703
0-1	30 (50.8)	16 (55.2)	
2-4	29 (49.2)	13 (44.8)	
Serum LDH level at diagnosis			0.825
Normal	46 (78.0)	22 (75.9)	
Elevated	13 (22.0)	7 (24.1)	
Serum β2-MG level at diagnosis			0.520
Normal	52 (88.1)	24 (82.8)	
Elevated	7 (11.9)	5 (17.2)	
Lesion at first registration			**0.007**
Single	42 (71.2)	12 (41.4)	
Multiple	17 (28.8)	17 (58.6)	
Invasion of deep intracranial areas			0.254
Yes	29 (49.2)	18 (62.1)	
No	30 (50.8)	11 (37.9)	
Pathological subtype			0.260
GCB	19 (32.2)	6 (20.7)	
Non-GCB	40 (67.8)	23 (79.3)	
Bcl-6			0.186
≥50%	44 (74.6)	18 (62.1)	
<50%	6 (10.2)	6 (20.7)	
unknown	9 (15.3)	5 (17.2)	
c-myc			0.625
≥40%	26 (44.1)	18 (62.1)	
<40%	15 (25.1)	8 (27.6)	
unknown	18 (30.5)	3 (10.3)	
MSKCC score (Risk)			0.628
1 (Low)	13 (22.0)	7 (24.1)	
2 (Intermediate)	38 (64.4)	16 (55.2)	
3 (High)	8 (13.6)	6 (20.7)	

RMA, rituximab + methotrexate + cytarabine; ECOG PS, Eastern Cooperative Oncology Group performance status; LDH, lactate dehydrogenase; β2-MG, β2-microglobulin; GCB, germinal B cell-like; MSKCC, Memorial Sloan−Kettering Cancer Center. Bold values: *P* < 0.05.

### Treatment responses

**Figure 2:**
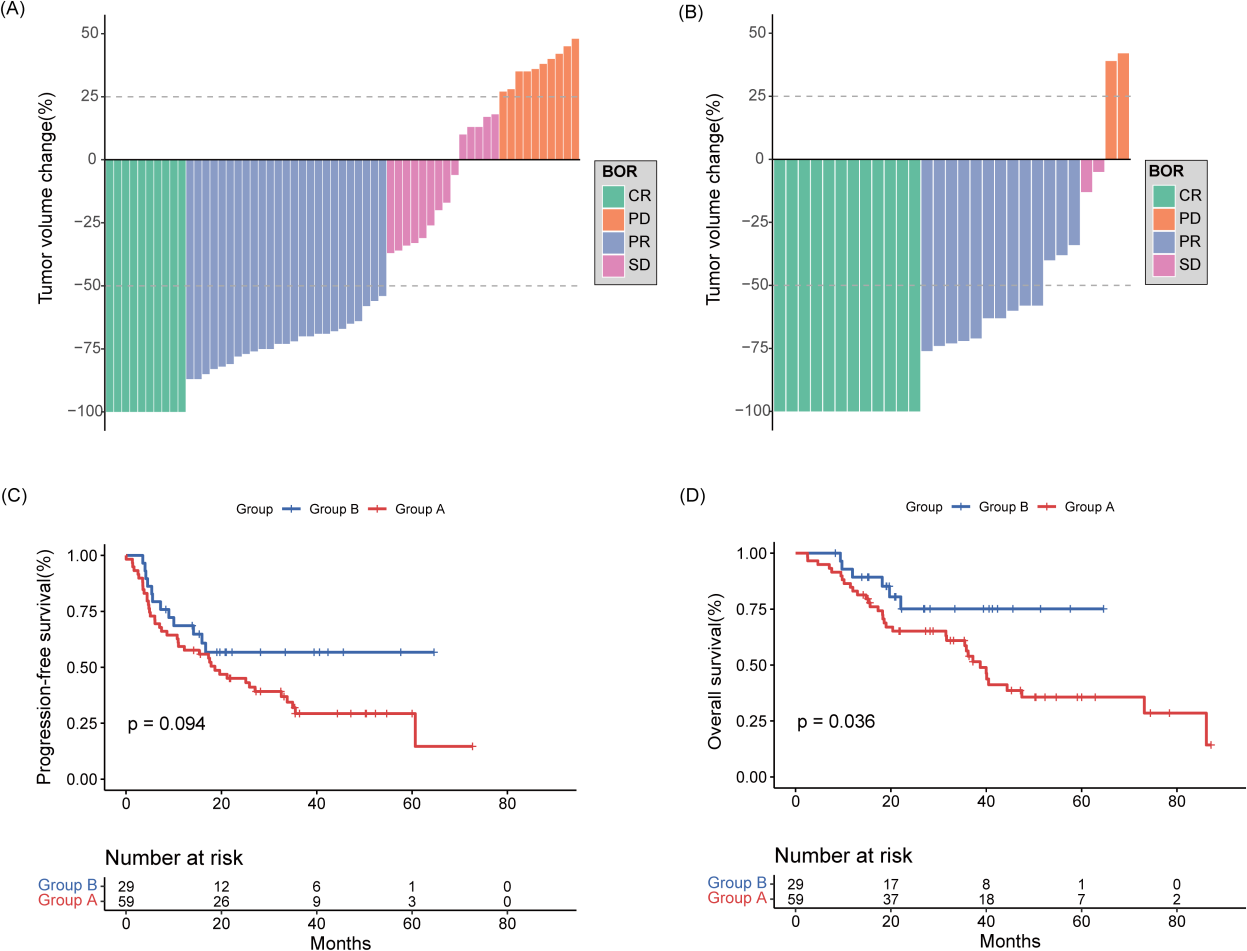
Best response to rituximab-methotrexate-cytarabine **(A)** and rituximab-methotrexate-cytarabine plus ibrutinib **(B)**. Percentage change of the total tumor volume from baseline was determined by MRI images. **(C)** Progression-free survival and **(D)** overall survival curves of collected patients divided according to induction treatment group.

All patients completed induction therapy. Twenty-five and 16 patients in Groups A and B underwent ASCT as consolidation therapy, respectively. Within Group A, 12 and 8 patients received lenalidomide and ibrutinib as maintenance therapies, respectively. In contrast, 18 patients in Group B were treated with ibrutinib as a maintenance therapy. Ten (16.9%) patients in group A and 12 (41.4%) patients in group B achieved a CR, with a significantly increased CR rate (CRR) in favor of group B, while 25 (42.4%) patients in group A and 13 (44.8%) patients in group B achieved a PR (**Figure 2**). The ORR was 59.3% in group A and 86.2% in group B, with a significantly increased ORR in favor of group B (**Table 2**). In a median follow-up of 27.7 (range 2.5–87.1) months of 88 patients, 36 (40.9%) patients were progression free, 19 (52.8%) of whom were in group A and 17 (47.2%) of whom were in group B. The 2-year PFS rates were 45.1% for group A, 56.7% for group B, and the 3-year OS rates were 56.3% for group A and 75.1% for group B. The median PFS and OS in group A were 18.6 (95% confidence interval (CI): 8.80-28.50) months and 38.7 (95% CI: 33.98-43.48) months, respectively. The median PFS and OS in group B were not reached (**Figure 2**).

**Table 2:** Response rate of group A and group B.

Response status	RMA (group A, n=59)	RMA+ Ibrutinib (group B, n=29)	*p* Value
CR	10 (16.9)	12 (41.4)	**0.013**
PR	25 (42.4)	13 (44.8)	0.827
SD	14 (23.7)	2 (6.9)	0.054
PD	10 (16.9)	2 (6.9)	0.323
ORR	35 (59.3)	25 (86.2)	**0.011**

RMA, rituximab + methotrexate + cytarabine; CR, complete response; PR, partial response; SD, stable disease; PD, progressive disease; ORR, overall response rate.

Bold values: *P* < 0.05.

**Figure 3:**
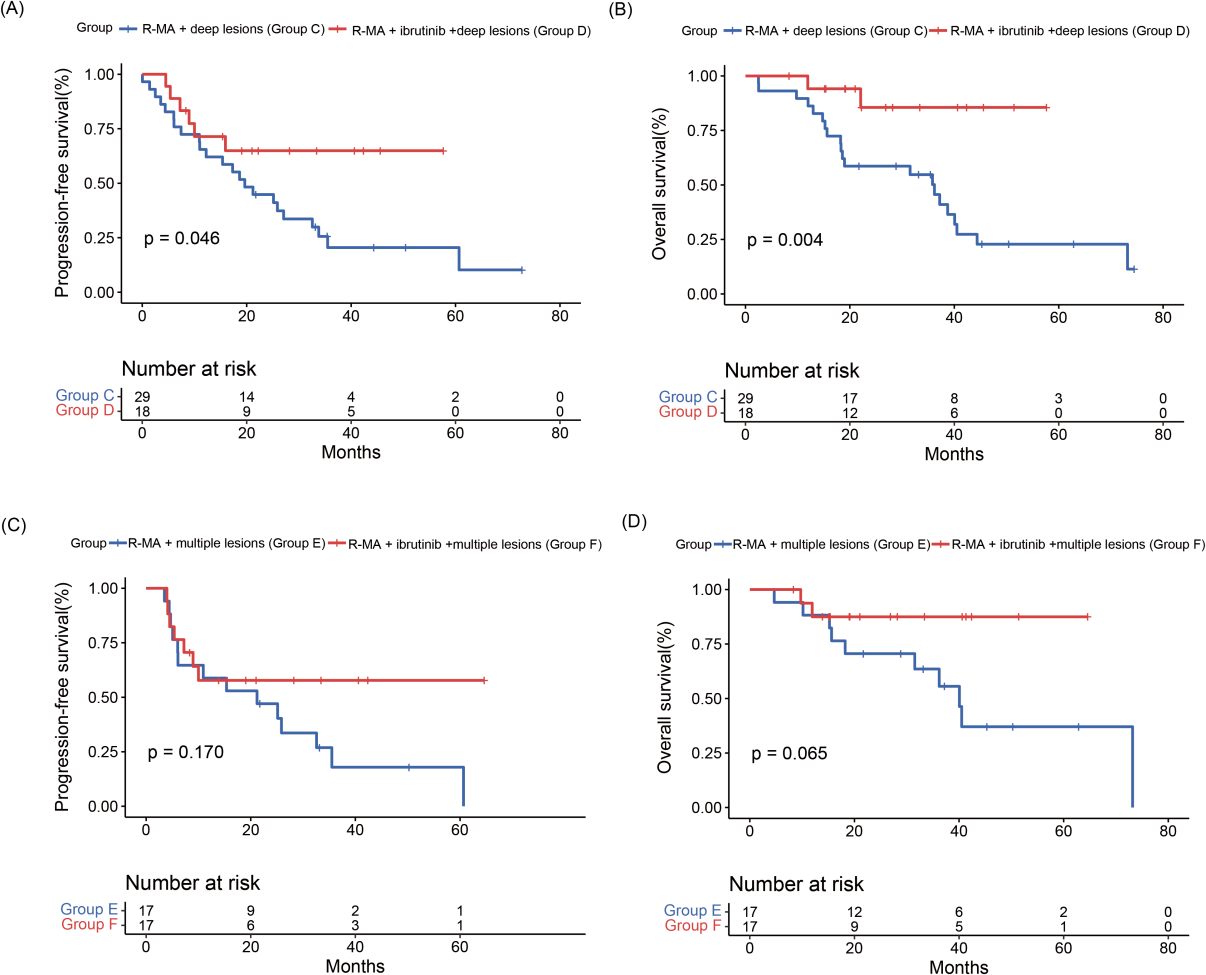
**(A)** Progression-free survival and **(B)** overall survival curves of patients in R-MA + deep lesions (group C) and R-MA + ibrutinib + deep lesions (group D). **(C)** Progression-free survival and **(D)** overall survival curves of patients in R-MA + multiple lesions (group E) and R-MA + ibrutinib + multiple lesions (group F).

Five (17.2%) patients in R-MA + deep lesions (group C) and 6 (33.3%) patients in R-MA + ibrutinib + deep lesions (group D) achieved a CR, while 12 (41.4) patients in R-MA + deep lesions (group C) and 10 (55.6%) patients in R-MA + ibrutinib + deep lesions (group D) achieved a PR. The ORR was 58.6% in R-MA + deep lesions (group C) and 88.9% in R-MA + ibrutinib + deep lesions (group D), with a significantly increased ORR in favor of R-MA + ibrutinib + deep lesions (group D) (**Supplementary Table S1**, **Supplementary Figure S1**). The 2-year PFS rate was 44.8% in R-MA + deep lesions (group C) and 64.9% in R-MA + ibrutinib + deep lesions (group D), with a median PFS of 19.63 (95%CI 12.77-26.49) months for R-MA + deep lesions (group C). The 3-year OS rate was 50.2% in R-MA + deep lesions (group C) and 85.6% in R-MA + ibrutinib + deep lesions (group D), with a median OS of 36.2 (95%CI 16.38-56.02) months for R-MA + deep lesions (group C). The median OS of R-MA + ibrutinib + deep lesions (group D) was not achieved. The PFS and OS in R-MA + ibrutinib + deep lesions (group D) were significantly better than those in R-MA + deep lesions (group C) (**Figure 3**).

Three (17.6%) of the 17 patients in R-MA + multiple lesions (group E) and 6 (35.3%) of the 17 patients in R-MA + ibrutinib + multiple lesions (group F) achieved a CR, while 7 (41.2%) of the 17 patients in R-MA + multiple lesions (group E) and 8 (47.1%) of the 17 patients in R-MA + ibrutinib + multiple lesions (group F) achieved a PR, with no significant difference between R-MA + multiple lesions (group E) and group F (**Supplementary Table S2**, **Supplementary Figure S1**). The 2-year PFS rates were 47.1% in R-MA + multiple lesions (group E) and 57.8% in R-MA + ibrutinib + multiple lesions (group F), with a median PFS of 21.20 (95%CI 2.98-39.43) months for R-MA + multiple lesions (group E). The 3-year OS rates were 63.5% in R-MA + multiple lesions (group E) and 87.5% in R-MA + ibrutinib + multiple lesions (group F), with a median OS of 40.1 (95%CI 33.86-46.28) months for R-MA + multiple lesions (group E). The PFS and OS of R-MA + multiple lesions (group E) and R-MA + ibrutinib + multiple lesions (group F) did not differ significantly (**Figure 3**).

### Adverse events

All 88 patients were included in the AE analysis. The more common AEs in group A and group B included hematological toxicities such as leukopenia (66.1% for group A, 55.2% for group B), anemia (69.5% for group A, 72.4% for group B), thrombocytopenia (64.4% for group A, 62.1% for group B) and nonhematological toxicities such as electrolyte imbalance (37.3% for group A, 44.8% for group B), hepatotoxicity (42.4% for group A, 31.0% for group B), and mucositis (18.6% for group A, 24.1% for group B) (**Table 3**). Accordingly, the most common hematological and nonhematological toxicities were anemia and electrolyte imbalance, respectively. Notably, nonhematological toxicities were mainly grade 1–2 and were usually mild. As expected for ibrutinib, grade 3–4 hepatotoxicity, electrolyte imbalance and digestive tract toxicity were more common in patients treated with R-MA + ibrutinib (group B) but cardiotoxicity was similar in the two groups. Grade 3–4 leukopenia, anemia and thrombocytopenia were increased in the R-MA group (group A) compared with those in the R-MA + ibrutinib group (group B). Treatment-related deaths were not observed in this study. After 2.5–87.1 months of follow-up, Thirty-nine patients died: 32 (82.1%) patients died from progressive disease, 3 (7.7%) from infection, 1 (2.6%) from renal failure, and 3 (7.7%) for unclear reasons.

**Table 3:** Main adverse events in group A and group B.

Adverse events	RMA (group A, n=59)			RMA+ Ibrutinib (group B, n=29)			*p* Value
Grade	0	1-2	3-4	0	1-2	3-4	
Hematological							
Leukopenia	20 (33.9)	22 (37.3)	17 (28.8)	13 (44.8)	10 (34.5)	6 (20.7)	0.287
Anemia	18 (30.5)	31 (52.5)	10 (16.9)	8 (27.6)	17 (58.6)	4 (13.8)	0.984
Thrombocytopenia	21 (35.6)	24 (40.7)	14 (23.7)	11 (37.9)	12 (41.4)	6 (20.7)	0.761
Non-hematologic							
Hepatotoxicity	34 (57.6)	23 (39.0)	2 (3.4)	20 (69.0)	7 (24.1)	2 (6.9)	0.400
Nephrotoxicity	48 (81.4)	9 (15.3)	2 (3.4)	22 (75.9)	6 (20.7)	1 (3.4)	0.565
Electrolyte imbalance	37 (62.7)	17 (28.8)	5 (8.5)	16 (55.2)	9 (31.0)	4 (13.8)	0.437
Mucositis	48 (81.4)	9 (15.3)	2 (3.4)	22 (75.9)	6 (20.7)	1 (3.4)	0.565
Digestive tract toxicity	40 (67.8)	17 (28.8)	2 (3.4)	21 (72.4)	6 (20.7)	2 (6.9)	0.753
Infection	48 (81.4)	6 (10.2)	5 (8.5)	26 (89.7)	1 (3.4)	2 (6.9)	0.346
Cardiotoxicity	57 (96.6)	2 (3.4)	0	27 (93.1)	2 (6.9)	0	0.460

RMA, rituximab + methotrexate + cytarabine.

### Relationships between clinical efficacy and gene mutations

**Figure 4:**
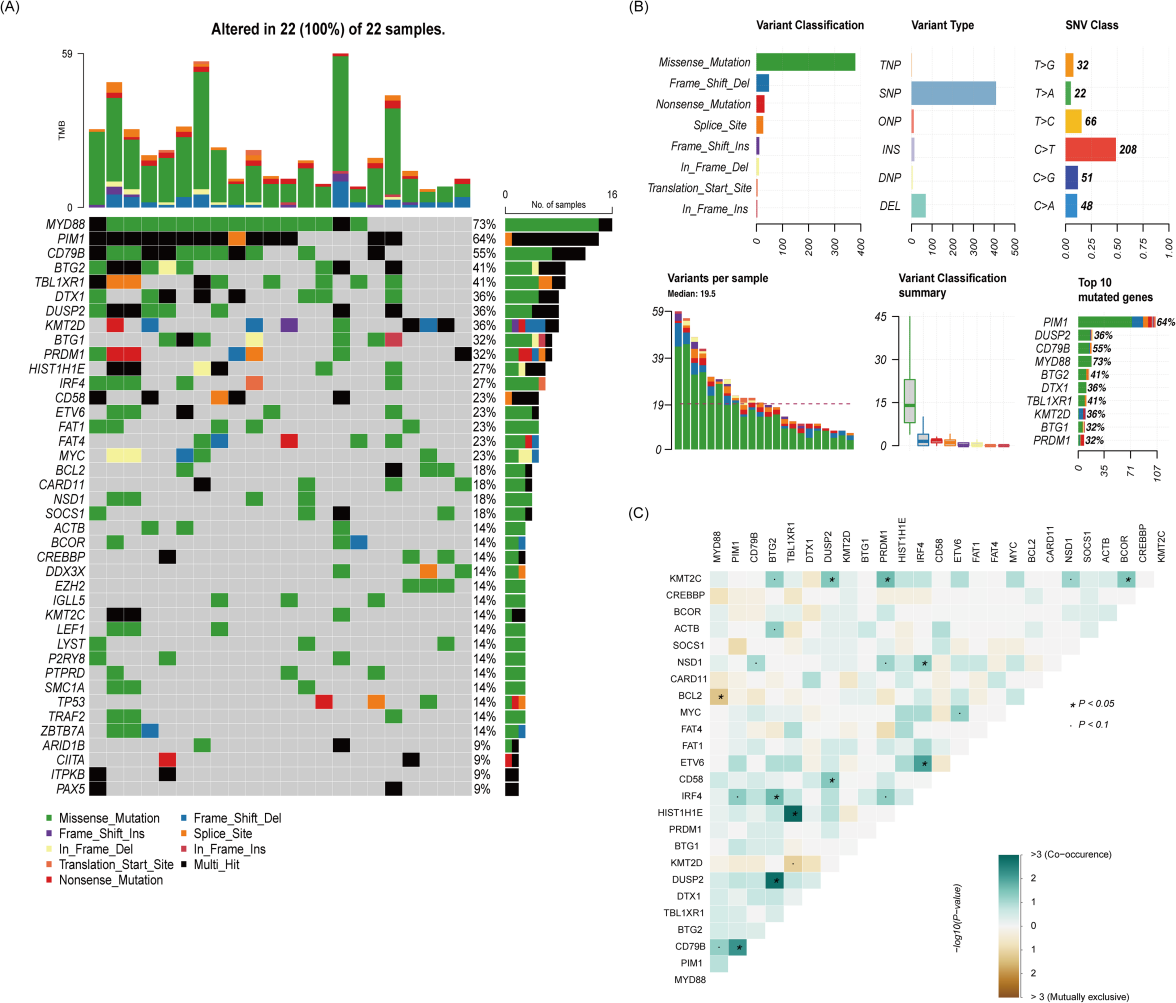
The relationship between clinical response and genetic characteristics in 22 patients treated with rituximab-methotrexate-cytarabine plus ibrutinib. **(A)** The gene mutation spectrum. **(B)** The whole picture of the mutation. **(C)** Analysis of gene co-mutation and mutual exclusion.

Sequencing data were available for twenty-two patients in group B, and the gene mutation status was shown in **Figure 4**. Of the 22 patients, 19 were the non-GCB subtype and 3 were the GCB subtype. *MYD88* (73%) was the most common mutation in primary tumor tissues, followed by the mutation of *PIM1*(64%) and *CD79B* (55%). The predominant type of mutation is of the missense type, and the variant type was mainly single-nucleotide polymorphism (SNP). Among the patients with *MYD88* mutation and *CD79B* mutation, two patients had *CARD11* mutation simultaneously, and these two patients did not respond to treatment. The remaining patients with *MYD88* mutation and *CD79B* mutation all had remission after treatment, and the two patients with *CARD11* mutation did not respond to treatment. Notably, the three patients without an *MYD88* or *CD79B* had response after treatment.

## Discussion

In recent years, the treatment of PCNSL has developed, and HD-MTX is the backbone of first-line treatment. Multi-drug chemoimmunotherapy regimens containing HD-MTX are considered to have better efficacy. Many researchers have suggested that ibrutinib has better efficacy and safety in patients with relapsed/refractory (R/R) PCNSL (19, 20, 22). However, studies on ibrutinib in newly diagnosed PCNSLs are rare. Therefore, we retrospectively analyzed the efficacy and safety of RMA and RMA plus ibrutinib regimens in newly diagnosed PCNSL patients. The results suggested that the RMA plus ibrutinib regimen increased the CRR, ORR and OS of newly diagnosed patients, and these regimens have better safety profiles in this patient population. More importantly, we found that RMA plus ibrutinib regimen may have better efficacy in the treatment of patients with newly diagnosed PCNSL with invasion of deep intracranial areas.

Marion Alcantara et al. (23) conducted a phase IB/II clinical trial to assess the efficacy and safety of rituximab, methotrexate, procarbazine, vincristine, and prednisone (RMVP) in combination with ibrutinib or lenalidomide for newly diagnosed PCNSL. After four cycles of induction therapy, the ORR for the lenalidomide and ibrutinib groups were 76.9% and 83.3%, respectively. The study observed a total of four dose-limiting toxicities (DLTs): one case of aspergillosis and pneumocystosis, one case of catheter-related infection, and two cases of elevated alanine aminotransferase levels (23). A retrospective study evaluated the efficacy and mutational profiles of HD-MTX combined with zanubrutinib in nineteen newly diagnosed PCNSL patients, the ORR, 2-year PFS and 2-year OS rate were 84.2%, 75.6% and 94.1%, respectively (24). The ORR of nine patients with ASCT as consolidation therapy was 88.9%, and the ORR of 10 patients with zanubrutinib as maintenance therapy was 80%. Chen et al. (25). analyzed data from real-world experience in treating newly diagnosed PCNSL with HD-MTX plus ibrutinib; 9 of 11 (82%) patients achieved a CR or PR, 7 of 11 (64%) patients achieved a CR, and the therapeutic approach was well tolerated. In our study, adding ibrutinib to the RMA combination was correlated with significant increases in the CRR, ORR and OS but not in PFS. The possible reasons for the tendency of the PFS curves to separate are as follows: first, the number of patients in the two treatment groups was inconsistent, and the number of patients in the whole cohort was small. Second, ibrutinib has been available for clinical use only recently, resulting in a limited follow-up time for patients in the RMA plus ibrutinib group. In this study, the ORR of RMA combined with ibrutinib group was 86.4%, and the 2-year PFS and 3-year OS rates were 56.7% and 75.1%, respectively, similar to previous studies. Notably, the cytarabine dose in this study (1 g/m²/day × 2 days) is lower than the IELSG32 trial (2 g/m²/day × 2 days) (9). The CRR and ORR in the R-MA group (16.9% and 54.2%) are notably inferior to those in IELSG32’s Arm B (CRR: 30, ORR: 74%). The reduced cytarabine dose possibly contributed to inferior efficacy and poorer outcomes. Several potential factors such as patient selection and supportive care also influence the efficacy and clinical outcomes. Most adverse events in our study were grade 1–2 and mild, consistent with findings of previous studies. More importantly, compared with the RMA regimen, the addition of ibrutinib to RMA was associated with similar toxicities. It is worth noting that, despite similar overall incidence of adverse events between the two groups, the incidence of grade 3–4 hematologic toxicities was higher in the R-MA group (group A) compared to the R-MA + ibrutinib group (group B) (leukopenia, 28.8% vs 20.0%; anemia, 16.9% vs 13.8%; thrombocytopenia, 23.7% vs 20.7%). This suggests that the addition of ibrutinib to the R-MA regimen primarily induces Grade 1–2 hematologic adverse events, which are generally well-tolerated by patients. Those results indicated that RMA plus ibrutinib regimen can increase the CRR, ORR and OS in patients with newly diagnosed PCNSL without additional toxicity.

The IELSG (26) score and Memorial Sloan Kettering Cancer Center (MSKCC) (27) prognostic score are typically used to stratify and evaluate the prognosis of PCNSL. The IELSG score consists of five factors, namely, age, ECOG PS, LDH level, cerebrospinal fluid (CSF) protein level and deep brain invasion, and each factor is worth one point. A score of 0 to 1 corresponds to low risk, 2 to 3 corresponds to intermediate risk, and 4 to 5 corresponds to high risk. The stratification of low, intermediate, and high risk correlates with 2-year survival rates of 80%, 48%, or 15%, respectively (28). The MSKCC score distinguishes three groups according to two factors: age and Karnofsky performance status (KPS). The median OS of PCNSL patients with age ≤ 50 years, age > 50 years and KPS ≥ 70, age ≥ 50 years and KPS < 70 were 8.5, 3.2 and 1.1 months, respectively (27). We analyzed the efficacy of R-MA+ deep lesions (group C) and R-MA+ ibrutinib + deep lesions (group D) and found that R-MA+ ibrutinib + deep lesions (group D) could significantly improve the ORR, PFS and OS of patients. In addition, the comparison of characteristics between the group A and group B revealed a more significant proportion of newly diagnosed PCNSL patients with multiple lesions in the group B. Therefore, we further compared the efficacy of R-MA+ multiple lesions (group E) and R-MA+ ibrutinib + multiple lesions (group F) and found that neither the response rates (CR rate, PR rate and ORR) nor the PFS and OS differed significantly between the groups. The possible reason for these negative results is the small number of group patients. However, the above results suggest that combining of RMA and ibrutinib increases the response rates and improves the prognosis of newly diagnosed PCNSL with adverse prognostic factors, such as deep brain involvement. Furthermore, no adjustment for multiple comparisons was performed despite numerous subgroup analyses including deep lesions and multiple lesions, thus the results of the subgroup analyses are exploratory. A large sample and prospective study should be carried out to explore this issue.

In recent years, studies on pathomechanistic genomic alterations have made significant progress. Genomic studies indicate that the emergence of lymphoma is driven mainly by disorders of the TLR, BCR, JAK-STAT and NF-κB signaling pathways, resulting in NF-κB inactivation (29, 30). In addition, the most commonly altered genes in the TLR and BCR signaling pathways are *MYD88*, *CD79B* and *CARD11* (31, 32). Therefore, upstream and downstream inhibitors of NF-κB, such as BTK inhibitors, are considered to inhibit BTK (the important element of BCR signaling) (19, 20). Currently, the molecular mechanisms of known resistance to ibrutinib in CNSL remains insufficiently understood. *CARD11*, a down-stream component of the BCR pathway, has been correlated with resistance to ibrutinib in B-cell malignancies (33, 34). A real-world study of ibrutinib combination therapy in the treatment of newly diagnosed PCNSL was conducted by Chen et al. (25), and the results showed that 9 patients who achieved ORR had mutations in BCR pathway genes (*MYD88*, 77.8%; *CD79B*, 33.3%; *CARD11*, 33.3%). Three patients with *CARD11* mutations also responded to ibrutinib combination therapy. A phase Ib clinical trial was initiated to explore the combination of ibrutinib with HD-MTX and rituximab in patients with CNSL (18). Twelve of 15 (80%) CNSL patients had mutations in ≥1 BCR pathway member (*MYD88*, 53%; *CD79B*, 47%; *CARD11*, 40%; *TNFAIP3*, 7%), 4 of 5 (80%) patients with *CARD11* mutations achieved responses after ibrutinib-based treatment. In addition, patients without detectable mutations in members of the BCR pathway still responded to ibrutinib-based treatment. In our study, fourteen of twenty-two (63.6%) PCNSL patients with a *MYD88* and (or) *CD79B* mutation responded to treatment, whereas four of twenty-two (18.2%) patients with a *CARD11* mutation did not respond to treatment. Therefore, whether *CARD11* is the main factor of ibrutinib resistance still needs further exploration and analysis. Notably, three of twenty-two (13.6%) patients with wild-type *MYD88* and *CD79B* in this study also had remission after treatment, which is consistent with the findings of Chen et al. (25). In PCNSL patients with wild-type *MYD88* and *CD79B*, several mechanisms may still allow for a response to ibrutinib. First, other signaling pathways may compensate for the lack of *MYD88* and *CD79B* mutations. For example, the NF-κB pathway, which is often activated downstream of BCR signaling, may still be active through alternative mechanisms (33). This can lead to ibrutinib sensitivity even in the absence of the typical *MYD88*/*CD79B* mutations. Second, ibrutinib is known to inhibit not only BTK but also other kinases such as ITK and TEC (33). These additional targets may contribute to its therapeutic effect in PCNSL patients, independent of *MYD88* and *CD79B* mutations. In addition, other factors, such as the specific subtype of PCNSL or the characteristics of individuals, may play a role.

We provide *post hoc* power calculation for PFS and OS. For the PFS endpoint, the ORR of RMA + ibrutinib (group B) was better than that of RMA (group A) (*P*=0.01), and the ORR of RMA + ibrutinib + deep lesions (group D) was superior to that of RMA + deep lesions (group C) (*P* < 0.05). For the OS endpoint, the ORR was similarly higher in RMA + ibrutinib (group B) than in RMA (group A) (*P*=0.01). However, there was no significant difference in ORR between RMA + deep lesions (group C) and RMA + ibrutinib + deep lesions (group D) (*P* > 0.05). The results demonstrated that this study was adequately powered to detect clinically meaningful differences.

In general, this study has several limitations. First, our study is a retrospective and small cohort study, and the small number of cases in the subgroup analysis led to significant negative differences between the therapeutic groups. And the treatment allocation was non-randomized, potentially introducing selection bias such as group B had more patients with multiple lesions (*P*=0.007). This baseline imbalance can mask the true differences in efficacy between RMA (group A) and RMA + ibrutinib (group B). Even if a treatment has potential benefits for all patients, the presence of more severe patients in group B may make the efficacy differences between the groups appear less significant. Second, the follow-up time of the group B was shorter, and the follow-up times of the group A and group B differed. Third, sequencing data are available for twenty-two patients, and the data of some patients are incomplete. Therefore, only a simple descriptive analysis can be conducted on the basis of the sequencing data; it is impossible to analyze the whole genomic characteristics of PCNSL and the relationship with therapeutic effects owing to the absence of large-scale sequencing data.

In conclusion, our retrospective study indicated that the addition of ibrutinib to RMA can clinically benefit newly diagnosed PCNSL patients and may improve the prognosis of PCNSL with adverse prognostic factors. On the basis of these results, we propose several exploratory analyses, such as the possibility that patients without mutations in BCR pathway members may respond to ibrutinib-based combination therapy through other mechanisms. However, studies with many patients and prospective clinical trials are needed to confirm these findings.

## Data Availability

The raw data supporting the conclusions of this article will be made available by the authors, without undue reservation.

## Supplementary material

Supplementary Figure 1 Best response of patients in R-MA + deep lesions (group C) **(A)** or R-MA + ibrutinib + deep lesions (group D) **(B)**. Best response of patients in R-MA + multiple lesions (group E) **(C)** or R-MA + ibrutinib + multiple lesions (group F) **(D)**.
