# Correction: Clinical outcomes of newly diagnosed PCNSL treated with rituximab-methotrexate-cytarabine with or without ibrutinib: a retrospective study

**DOI:** 10.3389/fimmu.2025.1648156

**Published:** 2025-09-04

**Authors:** Wenhua Wang, Bingyi Wang, Yifei Sun, Lihua Qiu, Zhengzi Qian, Shiyong Zhou, Zheng Song, Wei Li, Xudong Zhang, Lanfang Li, Xianhuo Wang, Huilai Zhang

**Affiliations:** ^1^ State Key Laboratory of Druggability Evaluation and Systematic Translational Medicine, Department of Lymphoma, Tianjin Medical University Cancer Institute and Hospital, National Clinical Research Center for Cancer, Tianjin’s Clinical Research Center for Cancer, Tianjin, China; ^2^ Key Laboratory of Cancer Prevention and Therapy, The Sino-United States Center for Lymphoma and Leukemia Research, Tianjin, China; ^3^ Department of Oncology, The First Affiliated Hospital of Zhengzhou University, Zhengzhou, Henan, China

**Keywords:** central nervous system, lymphoma, ibrutinib, retrospective, methotrexate

In the published article, there was an error in the author list, and author “Xudong Zhang” was erroneously excluded. The corrected author list appears below.

“Wenhua Wang^1,2†^, Bingyi Wang^1,2†^, Yifei Sun^1,2^, Lihua Qiu^1,2^, Zhengzi Qian^1,2^, Shiyong Zhou^1,2^, Zheng Song^1,2^, Wei Li^1,2^, Xudong Zhang^3*^, Lanfang Li^1,2*^, Xianhuo Wang^1,2*^ and Huilai Zhang^1,2*^”

They should also have the affiliation: “^3^Department of Oncology, The First Affiliated Hospital of Zhengzhou University, Zhengzhou, Henan, China”.

In the published article, there was an error in the Copyright statement. The statement was incorrectly written as “**Copyright ^©^** 2025 Wang, Wang, Sun, Qiu, Qian, Zhou, Song, Li, Li, Wang and Zhang. This is an open-access article distributed under the terms of the Creative Commons Attribution License (CC BY). The use, distribution or reproduction in other forums is permitted, provided the original author(s) and the copyright owner(s) are credited and that the original publication in this journal is cited, in accordance with accepted academic practice. No use, distribution or reproduction is permitted which does not comply with these terms.” The corrected statement is “**Copyright ^©^** 2025 Wang, Wang, Sun, Qiu, Qian, Zhou, Song, Li, Zhang, Li, Wang and Zhang. This is an open-access article distributed under the terms of the Creative Commons Attribution License (CC BY). The use, distribution or reproduction in other forums is permitted, provided the original author(s) and the copyright owner(s) are credited and that the original publication in this journal is cited, in accordance with accepted academic practice. No use, distribution or reproduction is permitted which does not comply with these terms.”

In the published article, there was also several errors in the text. “Some patient data were obtained from the First Affiliated Hospital of Zhengzhou University, and due to carelessness, we did not mention this in the article”.

A correction has been made to **Materials and methods**, *Patients and treatment*, Paragraph 1. This sentence previously stated:

“From March 2013 to October 2022, 88 newly diagnosed PCNSL patients from Tianjin Medical University Cancer Institute & Hospital (TMUCIH) were retrospectively enrolled and analyzed.”

The corrected sentence appears below:

“From March 2013 to October 2022, 88 newly diagnosed PCNSL patients from Tianjin Medical University Cancer Institute & Hospital (TMUCIH) and the First Affiliated Hospital of Zhengzhou University were retrospectively enrolled and analyzed.”

The study was also approved by the institutional review board of the Tianjin Medical University Cancer Institute & Hospital and the First Affiliated Hospital of Zhengzhou University. However, due to carelessness, only the former was mentioned in the article.

A correction has been made to **Materials and methods**, *Patients and treatment*, Paragraph 1. This sentence previously stated:

“The study was approved by the institutional review board of the Tianjin Medical University Cancer Institute and Hospital.”

The corrected sentence appears below:

“The study was approved by the institutional review board of the Tianjin Medical University Cancer Institute & Hospital and the First Affiliated Hospital of Zhengzhou University.”

The original article has been updated.

