# Supplementary figures and images for: Clinical outcomes of newly diagnosed PCNSL treated with rituximab-methotrexate-cytarabine with or without ibrutinib: a retrospective study
Source: Front Immunol. 2025 May 22;16:1579483. doi: 10.3389/fimmu.2025.1579483 (PMC12137331; doi:10.3389/fimmu.2025.1579483)

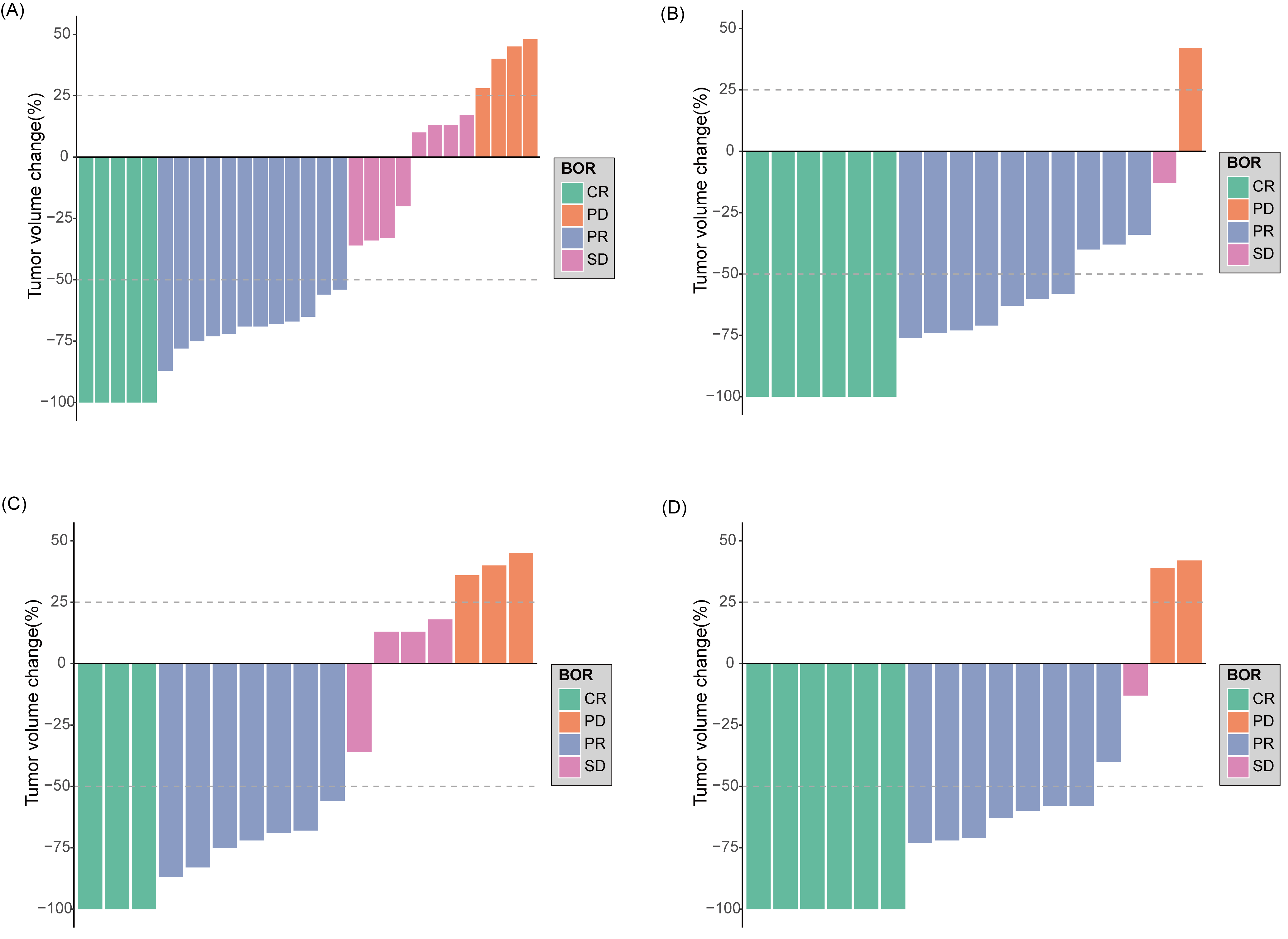

Supplement: Supplementary Figure 1 — Best response of patients in R-MA + deep lesions (group C) (A) or R-MA + ibrutinib + deep lesions (group D) (B). Best response of patients in R-MA + multiple lesions (group E) (C) or R-MA + ibrutinib + multiple lesions (group F) (D). [file Image1.tif]
