# Supplementary material for: Clinical outcomes of newly diagnosed PCNSL treated with rituximab-methotrexate-cytarabine with or without ibrutinib: a retrospective study
Source: Front Immunol. 2025 May 22;16:1579483. doi: 10.3389/fimmu.2025.1579483 (PMC12137331; doi:10.3389/fimmu.2025.1579483)
Supplement: Supplementary file 2 [file Table1.docx]

**Supplementary Table S1.** Response rate of R-MA + deep lesions (group C) and R-MA + ibrutinib + deep lesions (group D).

| Response status | R-MA + deep lesions (group C, n=29) | R-MA + ibrutinib + deep lesions  (group D, n=18) | *p* Value |
| --- | --- | --- | --- |
| CR | 5 (17.2) | 6 (33.3) | 0.291 |
| PR | 12 (41.4) | 10 (55.6) | 0.344 |
| SD | 8 (27.6) | 1 (5.6) | 0.124 |
| PD | 4 (13.8) | 1(5.6) | 0.636 |
| ORR | 17 (58.6) | 16 (88.9) | **0.027** |

RMA, rituximab + methotrexate + cytarabine; CR, complete response; PR, partial response; SD, stable disease; PD, progressive disease; ORR, overall response rate.

**Supplementary Table S2.** Response rate of R-MA + multiple lesions (group E) and R-MA + ibrutinib + multiple lesions (group F).

| Response status | R-MA + multiple lesions  (group E, n=17) | R-MA + ibrutinib + multiple lesions  (group F, n=17) | *p* Value |
| --- | --- | --- | --- |
| CR | 3 (17.6) | 6 (35.3) | 0.438 |
| PR | 7 (41.2) | 8 (47.1) | 1.000 |
| SD | 4 (23.5) | 1 (5.9) | 0.335 |
| PD | 3 (17.6) | 2 (11.8) | 1.000 |
| ORR | 10 (58.8) | 14 (82.4) | 0.259 |

RMA, rituximab + methotrexate + cytarabine; CR, complete response; PR, partial response; SD, stable disease; PD, progressive disease; ORR, overall response rate.
